# Autoantibodies, antigen-autoantibody complexes and antigens complement CA125 for early detection of ovarian cancer

**DOI:** 10.1038/s41416-023-02560-z

**Published:** 2024-01-09

**Authors:** Chae Young Han, Jacob S. Bedia, Wei-Lei Yang, Sarah J. Hawley, Lindsay Bergan, Marika Hopper, Joseph Celestino, Jing Guo, Terrie G. Gornet, Antoninus Soosaipillai, Hailing Yang, Samantha D. Doskocil, Anna E. Lokshin, Beverly C. Handy, Eleftherios P. Diamandis, Richard G. Moore, Karen H. Lu, Zhen Lu, Karen S. Anderson, Charles W. Drescher, Steven J. Skates, Robert C. Bast

**Affiliations:** 1 Department of Experimental Therapeutics, The University of Texas MD Anderson Cancer Center, Houston, TX USA; 2 Biostatistics Center, Massachusetts General Hospital, Boston, MA USA; 3 Translational Research Program, Fred Hutchinson Cancer Center, Seattle, WA USA; 4 Biodesign Institute, Arizona State University, Tempe, AZ USA; 5 Department of Gynecologic Oncology and Reproductive Medicine, The University of Texas MD Anderson Cancer Center, Houston, TX USA; 6 Department of Laboratory Medicine, The University of Texas MD Anderson Cancer Center, Houston, TX USA; 7 Lurenfeld-Tanebaum Research Institute (LTRI) Sinai Health System, Toronto, ON Canada; 8 Departments of Pathology, Medicine, and Obstetrics and Gynecology, University of Pittsburgh Medical Center and University of Pittsburgh Cancer Institute, Pittsburgh, PA USA; 9 Department of Obstetrics and Gynecology, Wilmot Cancer Center, University of Rochester Medical Center, Rochester, NY USA

**Keywords:** Diagnostic markers, Biomarkers

## Abstract

**Background:**

Multiple antigens, autoantibodies (AAb), and antigen-autoantibody (Ag-AAb) complexes were compared for their ability to complement CA125 for early detection of ovarian cancer.

**Methods:**

Twenty six biomarkers were measured in a single panel of sera from women with early stage (I-II) ovarian cancers (*n* = 64), late stage (III-IV) ovarian cancers (186), benign pelvic masses (200) and from healthy controls (502), and then split randomly (50:50) into a training set to identify the most promising classifier and a validation set to compare its performance to CA125 alone.

**Results:**

Eight biomarkers detected ≥ 8% of early stage cases at 98% specificity. A four-biomarker panel including CA125, HE4, HE4 Ag-AAb and osteopontin detected 75% of early stage cancers in the validation set from among healthy controls compared to 62% with CA125 alone (*p* = 0.003) at 98% specificity. The same panel increased sensitivity for distinguishing early-stage ovarian cancers from benign pelvic masses by 25% (*p* = 0.0004) at 95% specificity. From 21 autoantibody candidates, 3 AAb (anti-p53, anti-CTAG1 and annt-Il-8) detected 22% of early stage ovarian cancers, potentially lengthening lead time prior to diagnosis.

**Conclusion:**

A four biomarker panel achieved greater sensitivity at the same specificity for early detection of ovarian cancer than CA125 alone.

## Introduction

Ovarian cancer is the leading cause of gynecologic cancer death in developed countries [1]. This year in the United States, approximately 19,880 women will be diagnosed with ovarian cancer and 12,810 will die from this disease [2]. Advances in cytoreductive surgery and combination chemotherapy have improved 5-year survival in patients with epithelial ovarian cancer, but the rate of cure has changed little over the last two decades, due in part to diagnosis at a late stage (III-IV) in 70–75% of cases [3]. Disease detected before apparent spread from the ovaries (stage I) has a five year survival rate of >90% [3]. When disease is limited to the pelvis (stage II), five-year survival rates approach 70%, but when disease has spread to the abdomen (stage III) or above the diaphragm (stage IV), the cure rate slips to less than 20%. Therefore, detection of a larger fraction of women at an early stage could significantly impact survival. Computer models suggest that detection of ovarian cancer in early stage (I-II) could improve rates of cure by 10–30% [4].

Two major screening trials – the United Kingdom Collaborative Trial of Ovarian Cancer Screening (UKCTOCS) [5, 6] and the Normal Risk Ovarian Cancer Screening Study (NROSS) in the United States [7]- have evaluated rising CA125 with the Risk of Ovarian Cancer Algorithm (ROCA) to prompt transvaginal ultrasound (TVS) and an abnormal TVS to prompt surgery. While two-stage screening in the UKCTOCS failed to reduce mortality [5], it was associated with only a modest shift to earlier stage disease from 24% in controls to 38% with two-stage screening [6] a difference of only 14%. The smaller NROSS trial [7] was not designed nor powered to measure differences in mortality but detected 70% of cases in stage I or II, a stage shift of 46% from the UKCTOCS control. Conversely, NROSS screening reduced late stage (III-IV) disease by 34% compared to UKCTOCS controls and by 30% compared to US SEER values [8]. Reduction in mortality may require a greater reduction in late stage disease or a greater shift to early stage. The degree of stage shift has correlated with reduction of mortality in screening trials for non-small cell lung and breast cancers. A 20% or greater reduction in late stage disease has been followed by a significant decrease in mortality in randomized trials of mammography while smaller reductions of 10% or less have not been followed by a significant decrease in morality [9, 10].

CA125 is elevated in sera from only 60–70% of early stage epithelial ovarian cancers [11, 12] and better sensitivity at a high specificity might be achieved by the addition of biomarkers that complement CA125 [13, 14]. During the last two decades, we and others have made substantial efforts to test >120 serum biomarkers including autoantibodies (AAb), antigens and antigen-autoantibody complexes (Ag-AAb) to increase sensitivity and to detect cases missed by CA125 [15, 16].

Circulating autoantibodies (AAb) against ovarian cancer associated antigens provide one of the most promising candidates to augment the sensitivity of CA125 and to improve lead time [15–17]. AAb can be generated in response to very small amounts of tumor associated antigens and might be detected before ovarian cancers become large enough to shed measurable levels of serum biomarkers. Consequently, AAb might be detected months to years before elevation of CA125. In addition, we confirmed that elevated levels of anti-TP53 autoantibodies can be detected in as many as 20–25% of patients with ovarian cancer and titers of anti-TP53 rise 8 months prior to CA125 and 22 months prior to diagnosis in patients where CA125 levels did not increase [18]. Several assays have been developed for anti-TP53 autoantibodies and it has not been clear which has the greatest sensitivity at high specificity. Several other autoantibodies have been reported to be associated with ovarian cancer [15, 17, 19, 20], but panels of autoantibodies have received relatively limited attention.

When autoantibodies develop, shedding of ovarian cancer associated antigens can form antigen-autoantibody complexes. We have found that serum levels of HE4 Ag-AAb complexes complement CA125 to improve the detection of early-stage (I-II) ovarian cancer from 63% with CA125 alone to 81% with the combination [21]. Some tumor associated antigens can complement CA125. We previously demonstrated that osteopontin (OPN) was elevated in 50% of 76 early stage (I-II) ovarian cancers and detected 14% of CA125 negative cases [22].

With the support of the National Cancer Institute Early Detection Research Network (EDRN), a collaborative study has been conducted to compare panels of autoantibodies, antigen- autoantibody complexes and ovarian cancer associated antigens. Investigators at the MD Anderson Cancer Center (MDACC), Fred Hutchinson Cancer Center (FHCC), Arizona State University (ASU), University of Toronto, and the Massachusetts General Hospital (MGH) have measured 26 different biomarkers and compared 5 different assays for anti-TP53 AAb using serum samples from patients with early and late stage ovarian cancer, benign pelvic masses and healthy controls. A panel of 4 biomarkers has been identified that improves sensitivity at high specificity over CA125 alone for early stage (I-II) disease. Three promising autoantibodies have also been found in patients with early stage disease that may increase lead time over CA125 for early detection.

## Methods

### Patient serum samples

Human sera were obtained preoperatively from patients undergoing surgery at the University of Texas MD Anderson Cancer Center (MDACC) and the Fred Hutchinson Cancer Center (FHCC) as well as from the NROSS trial conducted at 11 sites in the United States. Informed consent was obtained with standard IRB-approved protocols at the University of Texas M.D. Anderson Cancer Center (MDACC: LAB04-0687), Fred Hutchinson Cancer Center (FHCC: 4563), University of Rochester (IRB exempt) and through the NROSS trial (MDACC: NCT00539162). An EDRN sample set was assembled with sera from 64 patients with stage I-II ovarian cancer, 186 patients with stage III-IV ovarian cancer, 200 patients with benign pelvic masses and 502 normal healthy controls (Supplementary Tables S1 and S2). The entire EDRN set was randomly split 50:50 into a training set and an independent validation set. Control sera were obtained from women who did not develop cancer while participating in the NROSS trial coordinated by MDACC or were healthy donors at the FHCC. All sera were processed and separated with standard operating procedures on the day that blood was obtained. Aliquots were frozen and stored at -80^o^ C. Samples were re-aliquoted once, coded (blinded), and distributed frozen to EDRN collaborators at ASU, FHCC, and MDACC.

### CA125 II and HE4 antigen assays

Both CA125II and HE4 were assayed on the Roche ELecsys Cobas platform (Roche, Indianapolis, IN) in an MDACC Department of Pathology research laboratory under CLIA conditions. All assays were performed according to the manufacturer’s instructions. The values of >35 U/ml for CA125II and > 70 pmol/L for HE4 are considered positive, respectively.

### P53 autoantibody assays were performed using the following five different platforms

The *Roche ELecsys Cobas assay* was performed according to the manufacturer’s instructions in an MDACC clinical laboratory. The *MagPlex/xMAP bead-based indirect serological assay* was developed and performed at MDACC as previously described [18] Briefly, recombinant human wild-type TP53 (BD Biosciences, San Jose, CA) was coupled to microspheres using an xMAP antibody coupling kit (Luminex, Austin, TX). A suspension of TP53 antigen-microspheres was prepared by diluting the coupled microsphere stocks (1 ×10^6^ beads/mL) to a final concentration of 50 beads/μL in PBS. Aliquots (50 μL/well) of microsphere suspension were placed in each well of a 96-well microplate. Serum samples (2 μL) were diluted in 48 μL PBS. Standard curves for quantitating TP53 AAb were assayed with a TP53 autoantibody calibrator (p53 ELISA^PLUS^ Autoantibody Kit, Calbiochem, Gibbstown, NJ). Samples and standards were added to 96-well microplates and incubated with TP53-coupled microspheres for 1 h at room temperature (RT) with gentle shaking. Beads were washed twice with PBS containing 0.05% Tween 20 (PBST) using a magnetic plate separator for 1 min and the liquid was discarded. Microspheres were then incubated with biotinylated goat anti-human IgG for 30 min at RT in the dark with gentle shaking. Plates were washed twice with PBST and microspheres were incubated with Streptavidin-R-phycoerythrin (SAPE), for 30 min at RT, in the dark with gentle shaking. After a final wash, the beads were re-suspended in 100 μL PBS buffer and fluorescence was measured on the MAGPIX system (Luminex, Austin, TX) with a minimum of 50 beads read per well. Data were analyzed with xPONENT software version 4.3 (Luminex Corp., Austin, TX). The *Rapid Antigenic Protein In situ Display (RAPID) assay* was performed at ASU as previously described [23] with the following modifications. Briefly, 96-well detection plates coated with anti-GST antibody (GE Healthcare, Piscataway, NJ) were incubated overnight at 4 °C with blocking buffer (5% milk in PBST). GST-p53 fusion protein was expressed from plasmids encoding wild-type p53, in the expression vector pANT7_cGST using a mammalian cell free protein 1-step human In Vitro Translation Kit (IVTT) (ThermoFisher Scientific, Waltham, MA). For each well, 0.92 uL of Hela lysate expression mixture was generated in accordance with the product manual recommendation. While proteins were expressed over 90 min, sera were diluted 1:100 with 10% *E. coli* lysate mixed in 5% milk in PBS-T and blocked until use. Then, the GST-fused proteins were diluted 1:100 in blocking buffer and added to the GST coated 96 well plates at 100 μL/well and shaken at RT on a plate shaker to allow the protein to bind to the plate. Then, prior to adding the blocked samples, assay plates were washed 5 times with ~200 uL/well of PBS-T. Diluted sample (100 uL) was added to the plate and incubated for 1 h at RT on a shaker to allow binding of sample antibodies to the captured protein. Upon incubation, the plates were washed 5 times with PBST. To visualize the captured antibody protein complexes, secondary HRP conjugated goat anti-human IgG antibodies (Jackson ImmunoResearch Laboratories, West Grove, PA) were diluted 1:10,000 in blocking buffer and aliquots of 100uL were added to each well on the plates and incubated for 1 hour. Plates were washed again 5 times with PBST. For developing, 100 µL of ECL (Thermo Scientific, Waltham, MA) were added to each well. Relative luminescence units (RLU) were measured on a Glomax 96 Microplate Luminometer (Promega, Madison, WI) at a wavelength of 425 nm. Values were plotted as mean values. Relative luminescence unit (RLU) ratios were calculated using the RLU of a specific antigen divided by the RLU of the control GST-protein. The sensitivity and specificity of AAb levels were determined by applying a cutoff value of the mean of the controls plus two standard deviations. The *Meso Scale Discovery Assay (MSD)* was performed according to the manufacturer’s protocol with slight modification (Meso Scale Diagnostics, Rockville, MD) [24]. For the custom multiplexed 4-spot plates from Meso Scale Discovery (MSD), recombinant proteins including CTAG2 (LifeSpan BioSciences), p53 (LifeSpan BioSciences), CA125 (Meridian Life Science) and NUDT11 (TBD) were provided to MSD in PBS. Protein (10 ng) were printed per spot and plates were stored in 4 °C until use. Both assay plates and serum samples were blocked overnight at 4 °C; plates were blocked with 5% blocking buffer and serum was diluted 1:100 with blocking buffer (5% Milk in PBST with 10% *E. coli* Lysate). After blocking, 25 uL of the serum were placed into the wells and incubated at RT for 1 h, washed 5 times with PBST, and 25 uL Sulfo-Tag labeled anti-Human IgG detection antibody (Meso Scale Discovery, Rockville, Maryland) at 1 ug/mL was added for 1 h at RT. The plates were washed 5 times with PBST, and 150 uL of the reading buffer was added and read with the Sector Imager (Meso Scale Discovery). The *SeroMAP bead assay* was performed at FHCC as previously described [25]. To generate a fusion protein, the TP53 gene was synthesized and cloned into a vector for in vitro expression (pANT7_cGST from Arizona Biodesign Institute, Tempe, AZ) with LR recombinase (Invitrogen, Carlsbad, CA). The resulting expression vectors were used to generate C-terminal GST fusion proteins in vitro using the TNT T7 Coupled Reticulocyte Lysate System (IVTT, Promega, Madison, WI) per manufacturer’s protocol. To couple anti-GST antibody to Luminex microspheres, 12 μg of goat anti-GST polyclonal antibody (GE Healthcare, Piscataway, NJ) was coupled to 2.5 ×10^6^ SeroMAP microspheres (Luminex) according to the manufacturer’s protocol. To perform bead-based IVTT-autoantibody assays, IVTT mixture containing GST-p53 was combined with anti-GST microspheres (1.6 μL IVTT reaction per 5000 beads) and incubated for 2 h at RT. TP53 fusion-protein-coated beads (5000 per well) were then added to a 96-well filter plate and washed twice with PBS, containing 0.05% Tween 20 (PBS-T). Human serum samples were diluted 1:1000 in PBS with 1% BSA; 50 μL of each diluted sample was added to the each well and incubated overnight at 4 °C. p53 protein expression for each IVTT batch was confirmed by detection with mouse anti-GST monoclonal antibody clone 26H1 (Cell Signaling Technology, Danvers, MA) at 2.5 μg/mL followed by PE conjugated anti-mouse IgG (Jackson ImmunoResearch Laboratories, Inc., West Grove, PA) at 1.25 μg/mL. Wells were washed twice with PBS-T and goat anti-human IgG antibody (Jackson ImmunoResearch Laboratories) were added at 1 μg/mL for 30 min at RT followed by washing with PBST. Streptavidin PE (BD Pharmingen, San Jose, CA) was added to each well at a 1:1000 dilution, incubated for 30 min and then washed with PBST. Then, beads were resuspended in 125 μL PBS-B followed by measurement of fluorescence intensity using the Bio-Plex Array Reader (Bio-Rad, Hercules, CA).

### OPN immunoassays

Enzyme-linked immunosorbent assays (ELISA) for OPN were performed at MDACC using MILLIPLEX MAP human circulating cancer biomarker magnetic bead panel kits purchased from Millipore Sigma (Burlington, MA, USA). The median fluorescent intensity (MFI) was measured by the Luminex MAGPIX system. The data were acquired and analyzed by xPONENT software version 4.2 (Luminex)

### HE4 Ag-AAb complex assays

HE4 Ag-AAb assays were performed as previously described [21]. In brief, 10^6^ microspheres were coupled with 6 µg of recombinant human HE4 protein (ACRO Biosystem, Newark, DE) using an xMAP antibody coupling kit. For each assay, a suspension of HE4 antigen-conjugated microspheres was prepared by diluting the coupled microsphere stocks (10^6^ beads/mL) to a final concentration of 50 beads/μL in PBS, pH 7.0. Serum samples for assay (2 μL) were diluted with PBS. Diluted sera were added to 96-well polystyrene microplates and incubated with HE4-coupled microspheres for 1 h at RT with gentle shaking. Following washing two times with PBST buffer, microspheres were incubated with detection antibody: either biotinylated goat anti-human IgG for serum samples and biotinylated goat anti-mouse IgG for serially diluted samples of the HE4 autoantibody standard. The plates were covered to protect them from light for 30 min at RT with gentle shaking. Plates were washed twice with PBST buffer and microspheres were incubated with SAPE. After the final wash, beads were re-suspended in PBS and fluorescence were measured as previously described in TP53 xMAP assay.

### Specificity and power

To compare sensitivities, specificity was set at 98% amongst healthy controls to achieve at least a minimum benefit to harm ratio for an early detection of ovarian cancer program in an asymptomatic postmenopausal population such as UKCTOCS and NROSS enroll. The minimum acceptable benefit to harm ratio is set to at most ten screen indicated surgeries being performed to detect earlier than clinical detection one case of ovarian cancer. To ensure this level of 10% is exceeded, the target positive predictive value (PPV) in screen indicated surgeries is set at 20%. The annual incidence of ovarian cancer in (apparently) healthy postmenopausal women in the UK and US for postmenopausal women is conservatively 1 in 2500, requiring a screening procedure to increase the incidence by 500-fold to achieve a 20% PPV. Following a positive CA125 blood test, TVS reduced the false positive rate due to the blood test by 10-fold (UKCTOCS). Thus, a blood test needs to increase the incidence by 50-fold for the combination of blood test followed by TVS to have a combined effect of 500-fold. The 50-fold increase in incidence corresponds to a blood testing having a false positive rate of 2%, that is, a 98% specificity, the specificity chosen at which to compare sensitivity between marker panels. These are approximate calculations and assume a high sensitivity which was achieved in UKCTOCS with 84% sensitivity for screening of ovarian cancer.

Power for sensitivity comparisons derives from the size of the validation set of *n* = 125 ovarian cancer cases. The study was planned with this sample size to provide a power of 97.8% at α = 5% to detect an increase in sensitivity from 60% to 75%, and a power of 77.9% at α = 5% to detect an increase from 65% to 75% (rule out 60% or lower, or 65% or lower, respectively, if the true sensitivity is 75%). The actual increase was 13% which, in retrospect, had a power of 93.5% at α = 5%.

### Statistical analysis

Within each group of cases, benign ovarian disease, and healthy controls, a *t*-test compared biomarker concentration means (log scale) between the EDRN sample sets from FHCC and MDACC to determine if there were any differences due to the patient accrual site. Autoantibody measurements including anti-TP53 AAb with multiple assay platforms were dichotomized based on cutoffs that achieved 98% specificity in the combined data set. For assessing the performance of serum protein biomarkers individually or as a panel, random forest models with 2,000 trees were trained on the training set and sensitivity at high specificity estimated by applying the random forest model to the validation set. The increase in sensitivity at a fixed specificity determined the extent that adding biomarkers to CA125 significantly improved prediction. The permutation test with 10,000 permutations of the case-control status was used to compute *p* values for testing the differences in sensitivity at a high specificity – 98% specificity for healthy controls to achieve a 2% false positive rate, and 95% specificity for benign controls given the lower specificity required for a symptomatic patient. Each permutation was applied to the combined set, the set was randomly (50:50) split into a training set and validation set, the random forest model trained on the permuted training set, and the resultant random forest model’s performance calculated on the permuted validation set. The *p*-value is the percentile of the sensitivity of the random forest model trained on the original (non-permuted) training set and evaluated on the (non-permuted) validation set amongst the sensitivities from the 10,000 permutations. All statistical analyses were performed using R software version 3.5.1 (R Foundation for Statistical Computing, Vienna, Austria).

## Results

### The discovery serum panel was split randomly into training and validation panels

The EDRN discovery set consisted of sera from ovarian cancer patients who were diagnosed at MDACC and FHCC through the EDRN consortium. The total serum panel included 64 early stage (I-II) (MDACC:21, FHCC:43) and 186 late stage (III-IV) ovarian cancer cases (MDACC:107, FHCC:79), 200 benign pelvic masses (MDACC: 56, FHCC:144), and 502 age matched healthy controls (MDACC:131, FHCC:371). (Supplementary Tables S1 and S2). Data from these samples were then split randomly (50:50) into a training set and an independent validation set. The most promising biomarker classifier was identified in the training set and its performance was compared to CA125 alone in the validation set.

### Elevated serum levels of anti-TP53 AAb were detected in early and late stage ovarian cancer with the RAPID assay exhibiting the greatest sensitivity

We previously reported that TP53 AAb were increased in ovarian cancer and that levels could be elevated 8 months prior to increases in CA125 and 22 months prior to clinical diagnosis when CA125 was not elevated. Several platforms have been used to assay anti-TP53 AAb. To determine which assay platform exhibited the highest sensitivity at 98% specificity, we compared 5 different assays to measure anti-TP53 autoantibodies using the entire EDRN serum set. The assay platforms included Luminex xMAP (MDACC), RAPID (ASU), Meso Scale Discovery (MSD, ASU), Luminex SeroMap (FHCC), and the Roche Elecys Cobas (MDACC). At 98% specificity (Table 1), sensitivity for all stages ranged from 12 to 22% across the assays, with the RAPID assay (ASU) demonstrating the greatest sensitivity. RAPID detected 12% of early stage cases and 25% of late stage cases. The Roche Cobas platform had the next best performance, detecting 9% of early stage cases and 23% of late stage cases. MSD detected 8% of early stage cases and 21% of late stage cases. SeroMAP detected 9% of early stage cases and 12% of late stage cases. xMAP detected 8% of early stage cases and 15% of late stage cases. Using the RAPID assay to measure anti-TP53 AAb, anti-TP53 detected 2% of cases missed by CA125.

**Table 1:** Percent sensitivity of different anti-TP53 AAb assays in benign disease and early stage (I-II) and late stage (III-IV) ovarian cancers at 98% specificity in healthy controls evaluated in the entire EDRN serum set.

Assay	Benign	Early	Late	All cases
RAPID	0.5	12.5	25.3	22.0
MSD	0.5	7.8	21.0	17.6
SeroMAP	1.0	9.4	12.0	18.0
xMAP	0.5	7.8	15.1	13.2
Roche Cobas	0.0	9.4	22.6	19.2

### Elevated serum levels of anti-CTAG1 and anti-IL8 autoantibodies and HE4 antigen autoantibody complexes were also found in early stage ovarian cancer

Levels of 21 other AAb and Ag-AAb complexes were measured in the validation serum set (Table 2 and Supplementary Table S3) including the anti-CTAG1, CTAG2, IL-8, CA4, ICAM3, KSR1, NUDT11, NXF3, POMC, PVRB, STXL1, TRIM-39 and UHMK1 autoantibodies from ASU as well as the MUC16L, Chr7.422.20, Chr7.422.10, PON1, MAGEC1, XRP1_E1_V9.11 and P21.2020 AAbs from FHCC (Supplementary Methods). In addition, *E. coli* absorbed anti-IL-8 AAb and HE4 Ag-AAb complexes were assayed at MDACC. Of the 21 AAbs and 1 immune complex marker tested, 4 had sensitivities of 8% or more at 98% specificity for early stage cases including TP53 AAb, CTAG1 AAb, IL-8 AAb and HE4 Ag-AAb complexes (Supplementary Table S3). Anti-CTAG1 detected 8% of early stage cases and 19% of late stage cases at 98% specificity, including 3% of cases missed by CA125. Among the autoantibodies, anti-IL-8 AAb assayed at MDACC showed the greatest sensitivity by detecting 26% of early stage cases and 16% of late stage cases, provided that anti-*E. coli* antibodies were absorbed prior to assay (Table 2). HE4 Ag-AAb complexes were also elevated in 19% of early stage and 9% of late stage cases (Table 2).

**Table 2:** Percent sensitivity of serum biomarkers for benign disease and for early stage (I-II) and late stage (III-IV) ovarian cancer at 98% specificity in healthy controls evaluated in the EDRN validation serum set.

Ag/AAb	Biomarker/Sensitivity (%)	Benign	Early	Late	All cases
	TP53 (RAPID)	0.5	12.5	25.3	22.0
	IL-8	8.5	26.5	15.6	18.4
	CTAG1	3.0	7.9	19.4	16.4
AAb					
	HE4 Ag-AAb	3.0	18.8	8.6	11.2
	CA125	22.0	62.5	93.5	85.6
	HE4	0.0	40.6	86.0	74.4
Ag	OPN	4.0	12.5	22.6	20.0
	HK 6	1.0	6.3	46.7	36.3
	HK10	2.0	9.4	49.5	39.2

### Panels of AAb alone do not have adequate sensitivity for detecting ovarian cancer

Anti-TP53 and anti-CTAG1 each detected 2% of early cases missed by CA125 and the combination detected 3%. When the best random forest classifier from the panel of Anti-TP53, anti-CTAG1A, and anti-IL8 was identified in the EDRN training set, the classifier detected 22% of early stage cases compared to 12% for anti-TP53 alone (*p* = 0.013) at 98% specificity in healthy controls. While the three autoantibodies detected more early stage disease than the best single antibody, sensitivity of this antibody panel alone would detect fewer early stage cases than CA125 alone and would not be adequate.

### Serum CA125 and HE4 were elevated in early stage ovarian cancer with some complementarity

The combination of CA125 and HE4 [26–29] has received particular attention and also has been used in the risk of malignancy algorithm (ROMA) to distinguish malignant from benign pelvic masses [30, 31]. To determine the degree of complementarity between CA125 and HE4 we measured two established clinical markers using a Roche platform in the EDRN discovery and validation sets. Our previous reports had found that CA125 was elevated in > 60% and HE4 in > 40% of early stage (I-II) epithelial ovarian cancers. In the EDRN validation set, CA125 had a sensitivity of 62% and HE4 a sensitivity of 41% for early stage cases at 98% specificity (Table 2). Among early stage cases with normal CA125 in the entire EDRN serum set, 11% had elevated HE4.

### Serum osteopontin (OPN) was elevated in early stage ovarian cancer and complemented CA125

Serum OPN has been reported as a robust biomarker for ovarian cancer by several groups [13, 22, 32–34]. Similarly, human kallikreins 6 (HK6) and 10 (HK10) have been reported as elevated in early and late stage disease [35]. The degree of complementarity of these 3 antigens with CA125 for detecting early stage ovarian cancer has, however, not been adequately evaluated. Using the EDRN Validation serum set, OPN was elevated in 12% of early stage and 23% of late stage cases (Table 2). Among early stage cases with normal CA125 in the entire EDRN set, 39% had elevated OPN at 98% specificity. HK6 and HK10 were elevated in 6% and 9% of early stage cases, respectively (Table 2), but demonstrated little complementarity with CA125 overall or in the random forest analysis of the EDRN training set.

### HE4, HE4 antigen-autoantibody complexes, and osteopontin complement CA125 for detecting early stage (I-II) ovarian cancer

**Fig. 1:**
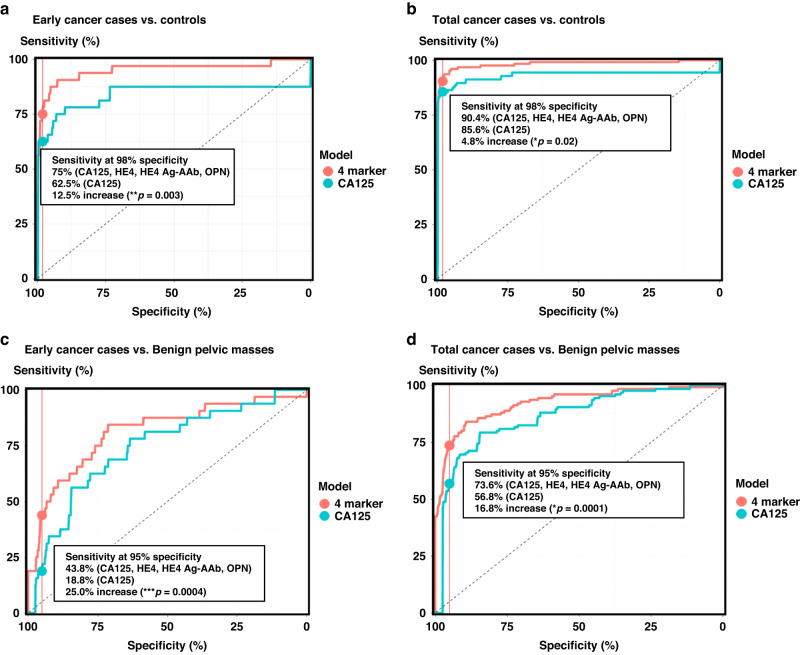
Receiver operating characteristic (ROC) curves of CA125 and 4- biomarkers for distinguishing ovarian cancer in the EDRN Validation serum set. Random forest based receiver operating characteristic (ROC) analysis compared the performance of CA125 with 4-biomarkers (CA125, HE4, OPN, and HE4 Ag-AAb) to distinguish: (**a**) early stage (I-II) cases from healthy controls at 98% specificity; (**b**) all ovarian cancer cases (I-IV) from healthy controls at 98% specificity; (**c**) early stage (I-II) cases from benign controls at 95% specificity (**d**) all ovarian cancer cases form benign controls at 95% specificity. Change in percentage sensitivity and *P* values are indicated in each panel.

Eight biomarkers each detected 8% or more of early stage ovarian cancer cases at 98% specificity in the EDRN data set. When these biomarkers were combined by the OR rule where a result is positive if any of the biomarkers are positive, a multi-biomarker panel of CA125, HE4, HE4 antigen-autoantibody complexes and osteopontin increased the sensitivity by 28% compared to CA125 alone, but at a substantially reduced specificity of 93%. To estimate the sensitivity of the 4-biomarker panel at 98% specificity, the EDRN data were split randomly (50:50) into training and validation sets. A random forest classifier defined on the training set detected 75% of early stage cancers in the EDRN validation set at 98% specificity compared to 62% sensitivity with a random forest classifier using CA125 alone at 98% specificity (*p* = 0.003) (Table 3), an increase of 13% in sensitivity for early stage ovarian cancer. The ROC curves show the validated sensitivities at 98% specificity and the significant improvement for early stage (*p* = 0.03) (Fig. 1A) and all cases (*p* = 0.02) (Fig. 1B).

**Table 3:** Percent sensitivity of a 4-biomarker panel (CA125, HE4, HE4 Ag-AAb, and OPN) compared to CA125 alone for detection of early stage (I-II) and late stage (III-IV) at 98% specificity in healthy controls from the EDRN Validation serum set.

Panel	Early	Late	Total
CA125, HE4, HE4 Ag-AAb, OPN	75.0	95.7	90.4
CA125	62.5	93.5	85.6
Delta	12.5**	2.2 (n.s.)	4.8*

Delta represents change in percent sensitivity between the 4-biomarker panel and CA125 alone.

*P < 0.05; **P < 0.01.

### Multi-marker panel better distinguishes between ovarian cancer and benign lesions

Distinguishing between a benign and malignant pelvic mass has clinical utility to help with the decision to refer the patient to a gynecologic oncologist before undertaking surgery [30]. To assess additional evidence for the complementarity of HE4, HE4 Ag-AAb, and OPN to CA125 in a multi-marker panel, random forest Classifiers were developed on the training set using either all four markers or CA125 alone to separate ovarian cancer patients from benign control subjects. Cases and benign controls in the EDRN training set were used to train the random forest classifier and the EDRN validation set was used to compare sensitivities at a 95% specificity, a specificity more appropriate for symptomatic patients and for distinguishing malignant from benign pelvic masses. At 95% specificity, the four-marker model achieved an overall sensitivity of 74% while the CA125 model had a sensitivity of 57% (*p* = 0.0001) (Table 4), an improvement in sensitivity of 17%. For early stage cases, the four marker random forest classifier had a 44% sensitivity compared to 19% sensitivity for CA125 alone at 95% specificity for benign controls, an improvement in sensitivity of 25% while keeping specificity fixed at 95% (*P* = 0.0004). These significant improvements in sensitivity for early stage cases and for all cases were found in ROC analysis (Fig. 1C, D, further demonstrating the complementarity and added value of HE4, HE4 Ag-AAb, and OPN to serum CA125 for detection of ovarian cancer).

**Table 4:** HE4, HE4 Ag-AAb, and OPN complement CA125 for early detection of ovarian cancer in benign controls from the EDRN Validation serum set at 95% specificity.

Panel	Early	Late	Total
CA125, HE4, HE4 Ag-AAb, OPN	43.8	83.9	73.6
CA125	18.8	69.9	56.8
Delta	25.0***	14.0**	16.8****

Delta represents change in percent sensitivity between the 4-biomarker panel and CA125 alone.

**P < 0.01; ***P < 0.001; ****P < 0.0001.

## Discussion

From previously published reports, CA125 can detect 60–70% of early stage (I-II) ovarian cancers at 97% specificity [12, 17]. In this study, serum CA 125 levels were elevated in 66% of early stage (I-II) ovarian cancers at 98% specificity in the EDRN Validation panel, leaving substantial room for improvement in sensitivity. A recent review identified 35 biomarkers that complement CA125 and could potentially enhance the sensitivity of a panel [17]. Particular attention has been given to panels that include both HE4 and CA125. In this study, HE4 detected 11% of cases missed by CA125 in the entire EDRN set. HE4 Ag-AAb complexes were elevated in 19% of early stage cases in the EDRN validation set (Table 2). Among the early stage cases with normal CA125 in the entire EDRN set, 28% had elevated HE4 Ag-AAb at 98% specificity.

In earlier studies, we had shown that OPN could be detected immunohistochemically in all of 65 CA125 negative ovarian cancers tested [13] and high levels could be found in sera from a significant fraction of patients with early stage disease. Using the EDRN validation set, we demonstrated that OPN was elevated in 13% of early stage ovarian cancers. Among the early stage cases with normal CA125 (<35 U/ml) in the entire EDRN set, 39% had elevated OPN at 98% specificity. OPN is a glycophosphoprotein that is secreted into body fluids by normal osteoblasts, arterial smooth muscle cells, different epithelial cells, activated T cells and macrophages, but can also be overexpressed by cancers that arise at different sites, including ovarian cancer [34, 36]

A classifier combining the biomarkers in the panel of CA125, HE4, HE4 Ag-AAb complexes and OPN was developed on the EDRN training set and tested on the EDRN validation set. CA125 alone detected only 62% of early stage cases, but the panel detected 75%, significantly improving detection (*p* = 0.003) by 13% (Table 3, Fig. 1A). A significant improvement in sensitivity (*p* = 0.02) was achieved for the entire EDRN validation set.

For biomarkers comparing ovarian cancer cases to benign controls, usually in symptomatic patients, the benefit to harm ratio has not been well defined and the incidence of cancer is much higher than in healthy controls. Both these aspects reduce the stringency for specificity, and we chose 95% specificity at which to compare sensitivities between marker panels. With further investigations into the minimum required benefit to harm ratio for this comparison, this choice of specificity could be revised.

A major goal of the EDRN collaboration has been to evaluate 20 AAb and 1 Ag-AAb complex (Table 2 and Supplementary Table S3), individually and as panels with a standard Discovery and Validation set from early and late stage ovarian cancer patients, as well as benign controls. Tumor-associated autoantibodies are produced during an immune response to overexpressed, mutant or post-translationally modified tumor associated proteins and may be increased by tumor-associated inflammation[37]. Conventional antigenic biomarkers such as CA125, HE4 and OPN may require a substantial tumor volume to raise serum levels above baseline observed in healthy women [38, 39]. From the literature, increased levels of at least 25 different autoantibodies have been associated with ovarian cancer [15, 16, 40], but few have been measured in large numbers of early stage ovarian cancers and showed distinguishable performance between cancer cases and benign cases.

Virtually all high-grade serous cancers are associated with mutated TP53 that complexes with wildtype TP53 and accumulates at high levels in the cytoplasm of ovarian cancer cells. Multiple studies have detected autoantibody to p53 in sera from ovarian cancer patients [18]. Our group had found that anti-p53 AAb were elevated in sera from 20 to 25% of ovarian cancer patients. In this study we compared 5 different assays to measure anti-TP53 AAb in the EDRN discovery set. The RAPID assay from ASU was the most sensitive, detecting TP53 AAb in 22% of all cases, but in only 12% of early stage ovarian cancers.

To detect a larger fraction of early stage cases, we evaluated 21 additional AAb and Ag-AAb complexes. Only anti-CTAG1 AAb and anti-IL8 AAb were elevated in a significant fraction (≥8%) of early stage (I-II) cases (Supplementary Table S3). CTAG1A detected 8% of early stage and 19% of late stage cases at 98% specificity. Anti-CTAG1 detected 3% of cases missed by CA125. Anti-IL-8 AAb showed the greatest sensitivity by detecting 16% of early stage cases. In the 18 early stage cases with normal CA125 (CA125 < 35 U/mL) in the EDRN discovery set, 7 (39%) had elevated anti-IL-8 AAb at 98% specificity. Anti-TP53 and anti-CTAG1 each detected 2% of early cases missed by CA125 and the combination detected 3%. When a classifier combining all three autoantibodies and complexes was trained in the EDRN training set, anti-TP53, anti-CTAG1 and anti-IL-8 detected 22% of early stage cases at 95% specificity compared to 12.5% for ant-TP53, the best single AAb (*p* = 0.013). The sensitivity of the three AAb did not exceed that of CA125 alone, indicating that sensitivity of this autoantibody panel used alone would not be adequate. Similarly, the addition of the 3 antibodies to the 4-biomarker panel (CA125, HE4, HE4 Ag-AAb complexes and OPN) did not increase sensitivity.

AAb can, however, produce lead time over CA125 that cannot be achieved with the other 3 biomarkers (HE4, HE4 Ag-AAb or OPN). In our previous studies, anti-TP53 AAb were detected 8 months prior to elevation of CA125 and 22 months prior to clinical diagnosis in patients who never experienced a rise in CA125 [18] in the UKCTOCS trial [5, 6]. Both anti-CTAG1 and anti-IL-8 have been detected >18 months prior to diagnosis in women destined to develop ovarian cancer, exhibiting sensitivities of 23% and 15% respectively at 98% specificity (Anna Lokshin, personal communication). Consequently, elevated titers of all 3 antibodies might provide lead time over CA125 and rise a year and a half prior to conventional diagnosis.

In conclusion, we have identified 3 biomarkers (HE4, HE4 Ag-AAb complexes and OPN) that complement CA125 and significantly increase the sensitivity for detection of early stage disease at 98% specificity in an independent validation set and three AAb (anti-TP53, anti-CTAG1 and anti-IL-8) that can detect 22% of early stage cases at 98% specificity and provide a potential increase in lead time of >18 months in a fraction of these cases. The challenge now is to demonstrate that the classifier based on the four-antigen panel and AAb have sufficient specificity to facilitate cost-effective screening for early detection of ovarian cancer with a clinical trial in the NROSS cohort.

### Supplementary information

Supplemental Material

## Data Availability

The data that support the findings of this study are available from the corresponding author upon request.
