# Supplementary material for: Autoantibodies, antigen-autoantibody complexes and antigens complement CA125 for early detection of ovarian cancer
Source: Br J Cancer. 2024 Jan 9;130(5):861–8. doi: 10.1038/s41416-023-02560-z (PMC10912308; doi:10.1038/s41416-023-02560-z)
Supplement: Supplementary file 1 — Supplemental Material [file 41416_2023_2560_MOESM1_ESM.docx]

**Supplementary Material**

**1. Supplementary Methods**

**2. Supplementary Table**

**3. References**

**Supplementary Methods**

**Anti-IL-8 autoantibody assays.** Magplex/xMAP indirect serological assays (Luminex Corp., Austin, TX) were performed to detect IL-8 AAb according to the xMAP Cookbook as previously described (1, 2) after absorbing with an extract of *E. coli* to remove antibodies reactive with the organism. Briefly, 3µg human recombinant IL-8 (R&D, Minneapolis, MN) was coupled to 10^6^ microspheres using an xMAP antibody coupling kit followed by confirmation of coupling efficiency with biotin-conjugated anti-human IL-8 (Thermofisher Scientific, Waltham, MA). A suspension of IL-8 antigen-microspheres was prepared by diluting the coupled microsphere stocks (10^6^ beads/mL) to a final concentration of 50 beads/μL in PBS buffer. Aliquots (25 μL/well) of microsphere suspension were placed in each well of a 96-well polystyrene microplate. Serum samples for assay (1 μL) were diluted in 24 μL of PBS with 1% *E. coli* suspension for a final protein concentration of 200 µg/mL. Serum samples were added to 96-well microplates and blocked with *E. coli* solution by incubating for 1 hour at room temperature (RT) with gentle shaking. A mouse anti-IL-8 monoclonal antibody (0.5µg/mL) (Origene, Rockville, MD) was used to set up a standard curve for quantitating the titer of IL-8 autoantibody. Prepared beads (25 uL) were mixed with a 25 uL serum sample blocked with *E. coli* as described above and incubated for 2 hours at RT. Beads were washed twice with PBS with 1% BSA using a magnetic plate separator for 1 min and the liquid was discarded by inverting the plate. Microspheres were then incubated with biotinylated goat anti-human IgG for 30 min at RT in the dark with gentle shaking followed by incubation with a SAPE for 30 min at RT. After washing 3 times with PBS, beads were re-suspended in 100 μL PBS buffer and fluorescence was measured on the MAGPIX system reading a minimum of 50 beads in each well. The data were acquired and analyzed by the xPONENT software version 4.3 (Luminex Corp., Austin, TX).

**Anti-CTAG1 and CTAG2 autoantibody assays.** CTAG1 and CTAG2 were measured by RAPID assays as described previously.

**PVR-B, TRIM-39, CA4, ICAM3, KSR1, NUDT11, NXF3, POMC, STXL1, UHMK1 autoantibody assays.** These AAb markers were measured with the RAPID/MSD methods at ASU as described previously.

**Anti-CHR7.422.10, CHR7.422.20, PON1.30, MAGEC1 and XRP1_E1_V9.11 autoantibody assays.** These AAb biomarkers were measured with a bead peptide-based assay at FHCC. 15-mer peptides were synthesized by Genscript (Piscataway, NJ) with N-terminal biotinylation to >85% purity. Peptides were coupled to Mag-Plex Avidin microspheres (Luminex) according to the Luminex XMAP Cookbook 4th Edition at a concentration of 1 µM. Peptide coated beads were added to a 96-well filter plate and washed as described previously. A 50 μl aliquot of 1:100 diluted serum was added to each bead-containing well and incubated for 30 minutes at RT with shaking at 800 RPM. Wells were washed 3 times with PBST and incubated with 1:500 diluted PE anti-human IgG (Jackson) for 30 minutes. Fluorescence intensity of washed, resuspended beads was measured using the Bio-Plex Array Reader (Bio-Rad, Hercules, CA).

**Anti-MUC16L autoantibody assays:** This marker was measured with a SeroMAP bead assay at the FHCC as described previously.

**Human Kallikrein 6 (HK6) and Human Kallikrein 10 (HK10) antigen assays.** Polyclonal anti-HK6 and anti-HK10 antibodies were generated as previously described. (3, 4) Serum samples were pre-diluted 10 times in buffer [6% BSA, 50mM Tris, pH:7.8]. To generate a sandwich-type, one-step immunofluorometric assay for HK6 and HK 10, white polystyrene microtiter plates were first coated overnight at room temperature with either monoclonal anti- HK6 (clone 27-4) and HK 10 antibodies 500ng/100ul/well anti-KLK10 (clone B14) monoclonal antibody diluted in a 50 mM Tris buffer (pH 7.80). The plates were then washed six times with washing buffer [containing 9 g/liter NaCl and 0.5 0.05% Tween 20 in 10 mM Tris buffer (pH 7.40)]. Standards or samples (100µl) were applied into each well. One hundred μL of another biotinylated monoclonal anti- HK6 or HK 10  antibodies diluted 1000-fold in assay buffer containing 6% BSA, 50 mM Tris , 0.05% sodium azide, 2.5% normal mouse serum, 10% normal goat serum, and 1% bovine IgG were also pipetted into each well (∼50 ng of antibody/well). The mixture was then incubated for 2 hours with shaking and washed with washing buffer for six times. Subsequently, the plates were incubated with 100 μL/well alkaline phosphatase-conjugated streptavidin (Jackson ImmunoResearch Laboratories, West Grove, PA), diluted 20,000-fold in a diluent containing 60 % BSA, 50 mMTris (, and 0.05% sodium azide for 15 min and washed. Finally, 100 μL of 1 mm diflunisal phosphate diluted in substrate buffer [0.1 m Tris (pH 9.1), 0.1 m NaCl, and 1 mm MgCl_2_) were added into each well and incubated for 10 min. Developing solution (100 µl, 1 m Tris base, 0.4 m NaOH, 2 mm TbCl_3_, and 3 mm EDTA) were pipetted into each well and mixed for 1 min. The fluorescence was measured with a time-resolved fluorometer, Envision 2103 Multilabel reader (Perkin Elmer).

**Supplementary Table S1. Contribution of UT MD Anderson (MDACC) and Fred Hutchinson Cancer Research Center (FHCC) sera to the entire EDRN panel**

| **Institution** | **Healthy Controls** | **Benign Pelvic Masses** | **Early (I/II)** | **Late (III/IV)** | **Ovarian Cancer** |
| --- | --- | --- | --- | --- | --- |
| **FHCC**  **(n = 637)** | 371 | 144 | 43 | 79 | 122 |
| **MDACC**  **(n = 315)** | 131 | 56 | 21 | 107 | 128 |
| **Total** | 502 | 200 | 64 | 186 | 250 |

**Supplementary Table S2. Clinical characteristics of ovarian cancer cases in the EDRN serum set**

|  |  |  | Age (years) | | Histology | |
| --- | --- | --- | --- | --- | --- | --- |
| Stage | No. | Ave | Median | Range | Type | No. |
| I | 36 | 55.2 | 57 | 23-81 | Serous | 17 |
|  |  |  |  |  | Endometrioid | 6 |
|  |  |  |  |  | Mucinous | 3 |
|  |  |  |  |  | Clear cell | 5 |
|  |  |  |  |  | Others | 5 |
| II | 28 | 61.3 | 58 | 45-85 | Serous | 18 |
|  |  |  |  |  | Endometrioid | 3 |
|  |  |  |  |  | Clear cell | 2 |
|  |  |  |  |  | Others | 5 |
| III | 164 | 59.7 | 60 | 36-85 | Serous | 155 |
|  |  |  |  |  | Endometrioid | 2 |
|  |  |  |  |  | Clear cell | 3 |
|  |  |  |  |  | Others | 4 |
| IV | 22 | 62.3 | 67 | 38-60 | Serous carcinoma | 20 |
|  |  |  |  |  | Others | 2 |
| Benign | 250 | 55.7 | 55 | 26-83 | N/A |  |
| Control | 502 | 60.9 | 54 | 28-88 | N/A |  |
| Total | 952 |  |  |  |  |  |

**Supplementary Table S3. Autoantibodies tested in the entire EDRN serum set at 98% specificity in healthy controls**

| **No.** | **Marker** | **Platform** | **Institute** | **Sensitivity (%)** | | | | **Sera Set** |
| --- | --- | --- | --- | --- | --- | --- | --- | --- |
|  |  |  |  | **Benign** | **Early** | **Late** | **Total** |  |
| **1** | **TP53 AAb** | **Roche Elecys** | **MDACC** | **0** | **9.4** | **22.6** | **19.2** | **Full set** |
|  | **TP53 AAb** | **Luminex Magpix** | **MDACC** | **0.5** | **7.8** | **21.0** | **17.6** | **Full set** |
|  | **TP53 AAb** | **RAPID** | **ASU** | **0.5** | **12.5** | **25.3** | **22** | **Full set** |
|  | **TP53 AAb** | **MSD** | **ASU** | **0.5** | **7.8** | **21.0** | **17.6** | **Full set** |
|  | **TP53 AAb** | **SERO** | **FHCC** | **1.0** | **9.4** | **12.0** | **18** | **Full set** |
| **2** | **CTAG1** | **RAPID/MSD** | **ASU** | **3.0** | **7.9** | **19.4** | **16.4** | **Full set** |
| **3** | **CTAG2** | **RAPID/MSD** | **ASU** | **2.5** | **7.9** | **24.7** | **15.6** | **Full set** |
| **4** | **IL-8 (*E. coli*)** | **Luminex Magpix** | **MDACC** | **8.5** | **26.5** | **15.6** | **18.4** | **Full set** |
| **4A** | **IL-8** | **RAPID** | **ASU** | **12.0** | **10.0** | **19.0** | **12.0** | **Full set** |
| **5** | **PVR-B** | **RAPID** | **ASU** | **3.5** | **4.8** | **6.0** | **5.6** | **Half set*** |
| **6** | **TRIM39** | **RAPID** | **ASU** | **2.0** | **6.3** | **3.8** | **4.4** | **Half set** |
| **7** | **CA4** | **RAPID** | **ASU** | **4.0** | **3.8** | **8.9** | **7.3** | **Half set** |
| **8** | **ICAM3** | **RAPID** | **ASU** | **5.2** | **0** | **1.6** | **1.2** | **Half set** |
| **9** | **KSR1** | **RAPID** | **ASU** | **7.6** | **6.7** | **5.1** | **5.3** | **Half set** |
| **10** | **NUDT11** | **RAPID** | **ASU** | **6.5** | **6.7** | **3.4** | **3.9** | **Half set** |
| **11** | **NXF3** | **RAPID** | **ASU** | **2.9** | **0.0** | **4.1** | **2.8** | **Half set** |
| **12** | **POMC** | **RAPID** | **ASU** | **3.3** | **1.0** | **0.0** | **0.7** | **Half set** |
| **13** | **STXL1** | **RAPID** | **ASU** | **2.9** | **0.0** | **4.1** | **2.8** | **Half set** |
| **14** | **UHMK1** | **RAPID** | **ASU** | **1.1** | **1.9** | **3.3** | **2.8** | **Half set** |
| **15** | **MUC16L** | **MSD** | **FHCC** | **2.2** | **0** | **4.1** | **3.3** | **Half set** |
| **16** | **Chr.7.422.20** | **bead based Peptide AAb** | **FHCC** | **3.3** | **6.7** | **4.9** | **5.2** | **Half set** |
| **17** | **Chr.7.422.10** | **bead based Peptide AAb** | **FHCC** | **2.2** | **6.7** | **1.6** | **2.6** | **Half set** |
| **18** | **PON1.30** | **bead based Peptide AAb** | **FHCC** | **2.2** | **6.7** | **1.6** | **2.6** | **Half set** |
| **19** | **MAGEC1** | **bead based Peptide AAb** | **FHCC** | **2.2** | **3.3** | **4.1** | **3.9** | **Half set** |
| **20** | **XRP1_E1_V9.11** | **bead based Peptide AAb** | **FHCC** | **2.2** | **3.3** | **2.4** | **5.2** | **Half set** |

- Some autoantibodies were assayed in half of the EDRN serum set and if <8% sensitivity for early stage disease was detected, not tested further.

**References**

1. Lokshin AE, Winans M, Landsittel D, Marrangoni AM, Velikokhatnaya L, Modugno F, et al. Circulating IL-8 and anti-IL-8 autoantibody in patients with ovarian cancer. Gynecol Oncol. 2006;102(2):244-51.

2. Hurley LC, Levin NK, Chatterjee M, Coles J, Muszkat S, Howarth Z, et al. Evaluation of paraneoplastic antigens reveals TRIM21 autoantibodies as biomarker for early detection of ovarian cancer in combination with autoantibodies to NY-ESO-1 and TP53. Cancer Biomark. 2020;27(3):407-21.

3. Luo LY, Katsaros D, Scorilas A, Fracchioli S, Bellino R, van Gramberen M, et al. The serum concentration of human kallikrein 10 represents a novel biomarker for ovarian cancer diagnosis and prognosis. Cancer Res. 2003;63(4):807-11.

4. Diamandis EP, Scorilas A, Fracchioli S, Van Gramberen M, De Bruijn H, Henrik A, et al. Human kallikrein 6 (hK6): a new potential serum biomarker for diagnosis and prognosis of ovarian carcinoma. J Clin Oncol. 2003;21(6):1035-43.
